# Calibration-free per-finger force-feedback slip control for grasping by anthropomorphic hand with tri-axial tactile sensors

**DOI:** 10.3389/frobt.2026.1735467

**Published:** 2026-02-09

**Authors:** Dickson Chiu Yu Wong, Zheng H. Zhu

**Affiliations:** Department of Mechanical Engineering, York University, Toronto, ON, Canada

**Keywords:** anthropomorphic hand, calibration-free, robotic grasping, slip detection, tactile sensors

## Abstract

This paper addresses the challenge of detecting and recovering from slip during robotic grasping of unknown objects, with the objective of establishing a robust no on-site or per-object calibration slip-recovery controller for an anthropomorphic hand. This hand is equipped with tri-axial piezoresistive tactile force sensors on each finger, and the proposed approach is validated through experimental analysis. The proposed methodology eliminates the need for object- or pose-specific calibration, explicit friction modelling, dense tactile arrays, line-of-sight vision, and a data-hungry learning process, enabling real-time implementation with minimal computation and integration effort. Using a commonly acquired online baseline from initial readings, slip is detected from relative changes between consecutive samples of the baseline-subtracted resultant tangential force, and object engagement is determined when the normal force reading deviates from a no-slip baseline beyond a preset threshold. Upon detecting slip, each finger increases its gripping force in closed-loop control until the slip stops, while enforcing motor-current protection in finger control to prevent actuator overload and object damage. Experiments were conducted on objects with different rigidity, weight, and surface textures, including an aluminium tube, a plastic water bottle, and a sponge. Additionally, the response time and variations in gripping force were evaluated. The results demonstrate rapid slip response via localized per-finger correction, good object conformability, and effective re-stabilization under different lifting speeds and sudden external disturbances. The per-finger design utilizes the minimum necessary correction at the offending finger, reducing unnecessary force increases on other fingers and improving grasp efficiency. This approach represents a practical solution for warehouse picking, human–robot collaboration, and *in situ* manipulation where task-specific calibrations, visual access, or training datasets are impractical.

## 1 Introduction

In prehensile manipulation, grasping is the foundational action upon which most manipulation behaviours are built, making it a central and active topic in the robotics community (Billard and Kragic, 2019). Its significance spans various domains, including industrial applications (Zhang et al., 2025a), agriculture (Wang et al., 2025b), food handling (Liu et al., 2023b), human-robot interactions (Ortenzi et al., 2021), and tasks involving delicate or unstructured environments (Jahanshahi and Zhu, 2024; Meng et al., 2022). In addition to grasp planning, the ability to sense and respond to slippage is equally crucial. The former establishes feasible and stable contact conditions, while the latter provides the real-time feedback necessary to maintain stability as conditions alter during operations. Grasp stability can be guaranteed by using geometric methods such as form closure or caging, which reduce the dependence on friction (Aceituno-Cabezas et al., 2023). However, these methods may not always work for unknown object geometry or pose, limited hand posture, or task constraints. In these situations, using reactive tactile feedback is a valuable complementary safety measure.

Nowadays, slip detection remains a fundamental challenge for both robotic grippers and anthropomorphic hands during grasping, particularly when manipulating unknown objects. Failure to quickly detect and correct slippage can lead to object loss, damage, and task failure (Romeo and Zollo, 2020). To address this issue, significant advancements have been made through the development of tactile sensors, which are configured either as standalone units or in array structures. These sensors can detect changes in electrical signals (Liu et al., 2023a; Yu et al., 2024), magnetic fields (Man et al., 2024), optical fiber (Mun et al., 2024), vibrations (Komeno and Matsubara, 2024), and convert them to useful force information for identifying slippage during grasping. Furthermore, visual-tactile sensors are another approach, in which the deformation of the elastomeric layer is translated into force information (Zhang et al., 2025b). However, several limitations exist. The need for calibration can complicate their use. Additionally, factors such as the frame rate of the camera, sensitivity to lighting conditions, and the heavy computational load of image processing can limit detection speed and affect the robustness and reliability of these sensors in slip detection applications.

Traditional slippage detection approaches depend on friction models (Pennestrì et al., 2016), which require prior knowledge of material properties and contact conditions, particularly the friction coefficient. The latter is typically absent for unknown objects. Transforming signals of contact forces from time to frequency domain provides another route (Romeo and Zollo, 2020; Qu et al., 2023), but it can introduce processing latency and may produce incorrect detections when the measurement is affected by non-slip vibrations, such as actuator motion and impacts. Recently, machine learning techniques have been employed to detect slip events directly from sensor data (Hu et al., 2023). However, they require substantial training data and adaptation to specific objects, limiting their generalizability across different tasks and contact conditions. Consequently, despite progress in both sensing and control, robust and scalable slip detection for everyday manipulation remains an open problem.

Current slip detection methods face several practical limitations. Regarding sensor design, there is a growing interest in array-based tactile skins. However, these often require dense sensor configurations and calibration processes for each sensor. For control strategies, model-based approaches often depend on measurements of normal and tangential forces, as well as known friction parameters, which are usually accompanied by the sensor calibration process. On the other hand, data-driven methods rely on large, specialized datasets tailored to specific sensors and tasks, along with significant computational resources. Moreover, both control approaches usually require prior knowledge of the objects being handled. Additionally, many of these solutions are designed primarily for parallel grippers or near-normal fingertip contact, rather than for the variable and oblique contact conditions found in anthropomorphic hands. What is lacking is a simple, calibration-free slip detection and recovery strategy that operates at the per-finger level, uses only low-dimensional tri-axial force measurements without vision or extensive learning, and remains robust when grasping unknown objects with varying poses and surface properties.

In this article, a closed-loop slip-detection method for an anthropomorphic hand during oblique fingertip contact is proposed, utilizing tri-axial piezoresistive tactile sensors. Referenced to a no-slip baseline estimated online from the initial readings, slippage is detected by the temporal variation of resultant tangential force computed from consecutive tri-axial tactile readings, and a consistent object contact is guaranteed by the normal force reading through a preset threshold value. Furthermore, motor-current protection is integrated directly into the same real-time control loop, improving stability without compromising actuator safety. This motivates the current study to develop a robust, calibration-free method for slip detection and recovery during the grasping of unknown objects using an anthropomorphic hand, equipped with tri-axial tactile force sensors on every finger.

## 2 Related works

### 2.1 Tactile sensing technologies

Recent reviews have surveyed advances in tactile sensing for human–robot interaction (Jassim et al., 2025) and in pushing and grasping manipulation in robotic arms (Efendi et al., 2025). In contact detection, piezoresistive sensors are widely used to detect contact onset and localize interaction points. Early studies have shown that soft sensor arrays are capable of producing real-time force maps with sub-millimeter precision, which is advantageous for localization in manipulation, establishing an example for feedback-driven grasp control (Hammond et al., 2014). Furthermore, recent work embeds piezoresistive networks directly into compliant robot hands and grippers, allowing them to maintain stable grasps when vision is unavailable, which includes fully 3D-printed wearable finger arrays for pressure-point localization and large-strain piezoresistive skins that also enable proprioceptive estimation and object classification (Pei et al., 2021; Yong et al., 2022). Moreover, in prosthetics, printable piezoresistive composites and skin-inspired tactile elements have been integrated to support safe interaction and haptic perception in unstructured environments (Lathers et al., 2017; Wu et al., 2018).

### 2.2 Control strategies for grip adjustment

On control strategies, piezoresistive sensors enable slip detection and closed-loop grip adjustment by capturing resistance fluctuations linked to shear forces. This capability has been demonstrated in both rigid and soft grippers, allowing real-time adaptation when handling fragile objects that require minimal force. Slip detection methods can be broadly categorized into three approaches. The traditional model approach utilizes thresholds or model-based rules that focus on normal force rate, tangential components, or friction models to identify incipient slip (Stachowsky et al., 2016; Liu and Howe, 2023; Deng et al., 2017). In contrast, electrical signal detection methods directly analyze electrical readouts from piezoresistive arrays, utilizing per-taxel voltage drops or current spikes during micro-slip as indicators (Chen et al., 2024; 2021). Changes in directional force, such as an increase in specific direction, can also indicate the onset of sliding (Zhang et al., 2015). These methods can be sensitive to contact orientation, as object contact at an oblique angle can introduce normal-shear coupling, which biases tangential force estimates. This confusion may lead to incorrect detection unless pose-aware compensation or decoupling techniques are applied. Slip has also been inferred from contact acceleration or vibration, which typically requires dedicated inertial sensing elements and higher-bandwidth sampling and processing (Howe and Cutkosky, 1989). Consequently, their applicability can be constrained by the complexity of sensor integration and their sensitivity to non-slip vibrations. Lastly, the learning approach leverages time-space patterns from sensor arrays to generalize across various objects and grasp types. The limitation here mainly concerns the need for large labelled datasets to effectively cover a variety of objects, surfaces, and angles during the learning process (Zhao et al., 2025; Wang et al., 2025a).

### 2.3 Applications in dexterous/anthropomorphic hands

On the application side, many studies focus on the positioning of the sensing surface parallel to the object, in which the sensing surface is placed parallel to the object by using either parallel grippers (Sui et al., 2024; Stachowsky et al., 2016) or anthropomorphic hands (Gong et al., 2021; Zhang et al., 2015; Deng et al., 2017) with fingertips with near-normal contact. However, in real-world grasping situations, each finger makes contact with the object at different oblique angles. Recently, some researchers have started using anthropomorphic hands equipped with tactile sensors in the fingertips, utilizing advanced machine learning techniques (Zhao et al., 2025; Chen et al., 2025). These developments highlight the need to create designs that can robustly adapt to pose variations and develop testing protocols that accommodate a range of contact angles.

## 3 Strategy and implementation for slip detection

### 3.1 Problem statement

Most tactile sensor-based slip detection methods are typically designed for parallel grippers, which assume that the contact between the fingertips and the surface of an object is nearly parallel. In contrast, anthropomorphic hands often make contact with an object at various angles across different fingers, creating a challenge for current slip detection systems. The proposed method addresses this issue by introducing a per-finger closed-loop slip controller. This controller uses force readings from a tri-axial piezoresistive force tactile sensor and infers slip from temporal changes in the resultant tangential force between consecutive readings, referenced to a runtime no-slip baseline. Therefore, the proposed controller avoids the need for explicit friction modeling, data-driven slip classifiers, inertial sensing elements, or frequency-domain processing. Here, calibration-free denotes that no additional user-performed calibration, object- or pose-specific tuning, or friction-parameter identification is required beyond the factory force output from the sensor and a short runtime baseline acquisition. This approach is designed to be robust against various uncertainties associated with the object, such as rigidity, weight, and surface textures. Additionally, the per-finger sensor configuration allows for independent slip detection for each finger. Control is only activated for the finger that detects a slip, which prevents excessive force from being applied by the other fingers on the object. This simple approach ensures robust slip detection with good object conformability while maintaining safe and stable grasp control.

### 3.2 Research methods

#### 3.2.1 Hardware setup

**FIGURE 1:**
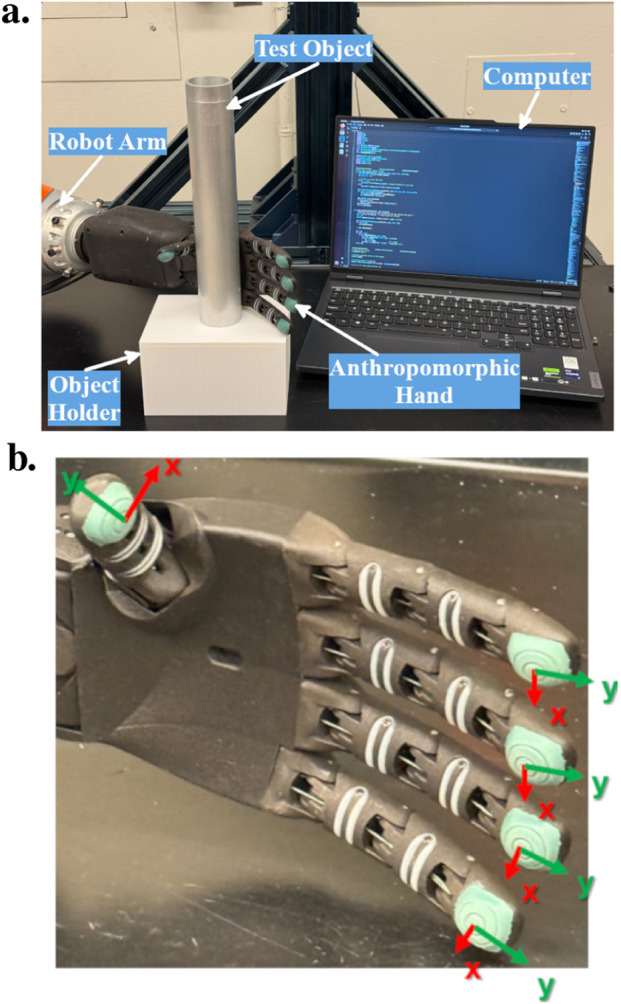
System setup for this study. **(a)** Overall system setup. **(b)** Anthropomorphic hand used in this study. The local coordinates of each tactile sensor are illustrated, with the z-axis pointing out of the page, which is not shown in the figure.

The experimental platform is shown in Figure 1a, which consisted of a 7-degrees-of-freedom (DoF) robot arm (LRB iiwa 14 R820, KUKA), equipped with a five-fingered, cable-driven anthropomorphic hand (RH8D, Seed Robotics), which has 8 DoFs. In practice, only the DoFs relevant for controlling the bending of the fingers were employed. Also, in the design of the hand, the ring and pinky fingers are actuated by a single motor, resulting in both digits sharing a single DoF. Therefore, a total of 4 DoFs were used in this study. Moreover, the motor controlling the finger bending provides 12-bit resolution. The finger is fully straight at motor position 0 and fully bent at motor position 4,095.

Each fingertip is equipped with a three-axis piezoresistive tactile sensor (FTS3, Seed Robotics) that exhibits a resolution of 1 mN and a force measurement range of 30 N, with a sampling frequency of 50 Hz. The sensor measures forces along the x, y, and z directions based on their respective local coordinates, as shown in Figure 1b. Real-time data were collected from sensors, allowing for the computation of various parameters for slip detection and grasp control. Given that the ring and pink fingers shared a motor, the average of their respective sensor readings was used to control the shared DoF.

#### 3.2.2 Research design

**FIGURE 2:**
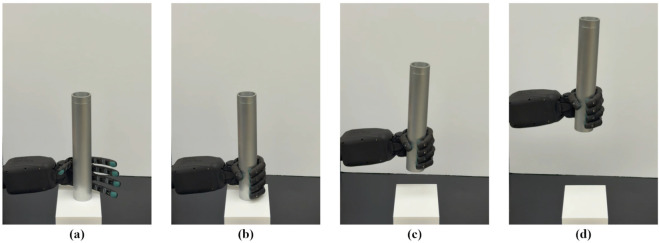
Experimental procedure: **(a)** Engaging the object. **(b)** Moving the robot arm upward to induce slip once all fingers touch the object. **(c)** Implementing slip control. **(d)** Achieving a stable grasp.

The slip detection control consists of four phases, as illustrated in Figure 2. In phase 1, the robot’s fingers move toward the object until they gently make contact with its surface, which is monitored by measuring the force in the z-direction. Once contact is established, phase 2 involves the robot arm lifting the object to create a potential slipping event. In Phase 3, the slip detection control is activated, during which three operations occur concurrently. The system maintains object engagement by continuously monitoring the z-directional force, detecting slip by analyzing changes in the resultant tangential force between consecutive readings, and ensuring circuit protection by monitoring the electrical currents of the motors. Based on these measurements, commands are sent to each motor individually to adjust the gripping force and prevent slippage, as demonstrated by a stable grasp in the final stage.

Algorithm 1 **Grasping and Slip Detection Control.**

**Require:** $f_{z}^{TH}$ , $f_{xy}^{TH}$ , $I_{\mathrm{motor}}^{TH}$ , $\delta f_{z}$ , $\delta f_{xy}$ , δI , $P_{\mathrm{motor}}^{TH}$

1: **function** Motor Position Adjustment( $f_{z}^{\mathrm{raw}}$ , $f_{z,0}$ , $P_{\mathrm{motor}}$ , $P_{\mathrm{motor}}^{TH}$ , $\delta f_{z}$ )
2:  Get $f_{z}^{\mathrm{raw}}$ , $P_{\mathrm{motor}}$

3:  Calculate $f_{z}^{\mathrm{control}}=abs\left(f_{z}^{\mathrm{raw}}-f_{z,0}\right)$

4:  **if** $\left(f_{z}^{\mathrm{control}}<f_{z}^{TH}\right)$ and $\left(P_{\mathrm{motor}}<P_{\mathrm{motor}}^{TH}\right)$ **then**
5:   Increase $P_{\mathrm{motor}}$ by $\delta f_{z}$

6:  **else**
7:   Store $P_{\mathrm{motor}}$ as the updated value
8:   **end if**
9: **end function**
  **Stage 1: Baseline Forces Acquisition Phase**
10: Record first 20 sensor readings
11: Take the average and store as $f_{x,0}$ , $f_{y,0}$ , $f_{z,0}$

12: Calculate $f_{xy,0}=\sqrt{{\left(f_{x,0}\right)}^{2}+{\left(f_{y,0}\right)}^{2}}$

  **Stage 2: Object Engagement Phase**
13: **while** true **do**
14:  Get $f_{z}^{\mathrm{raw}}$ , $P_{\mathrm{motor}}$

15:  Motor Position Adjustment( $f_{z}^{\mathrm{raw}}$ , $f_{z,0}$ , $P_{\mathrm{motor}}$ , $P_{\mathrm{motor}}^{TH}$ , $\delta f_{z}$ )
16:  Get $I_{\mathrm{motor}}$

17:  **if** $\left(P_{\mathrm{motor}}\;\geq \;P_{\mathrm{motor}}^{TH}\right)$ or $\left(abs\left(I_{\mathrm{motor}}\right)\;\geq \;I_{\mathrm{motor}}^{TH}\right)$ **then**
18:   Break
19:  **end if**
20:  **end while**
  **Stage 3: Slip Detection**
21: **while** true **do**
22:  Motor Position Adjustment( $f_{z}^{\mathrm{raw}}$ , $f_{z,0}$ , $P_{\mathrm{motor}}$ , $P_{\mathrm{motor}}^{TH}$ , $\delta f_{z}$ )
23:  Get $f_{x}^{\mathrm{raw}}$ , $f_{y}^{\mathrm{raw}}$ , $P_{\mathrm{motor}}$

24:  Calculate $f_{xy}=\sqrt{{\left(f_{x}^{\mathrm{raw}}-f_{x,0}\right)}^{2}+{\left(f_{y}^{\mathrm{raw}}-f_{y,0}\right)}^{2}}$

25:  **if** $f_{xy}$ is the first reading **then**
26: $\Delta f_{xy}=abs\left(f_{xy}-f_{xy,0}\right)$

27:  **else**
28: $\Delta F_{xy}=\;abs\left(f_{xy}\left(\text{current value}\right)-f_{xy}\left(\text{previous value}\right)\right.$

29:  **end if**
30:  **if** $\left(\Delta f_{xy}>\Delta f_{xy}^{TH}\right)$ and $\left(P_{\mathrm{motor}}<P_{\mathrm{motor}}^{TH}\right)$ **then**
31:   Increase $P_{\mathrm{motor}}$ by $\delta f_{xy}$

32:  **else**
33:   Store $P_{\mathrm{motor}}$ as the updated value
34:  **end if**
35:  Get $P_{\mathrm{motor}}$ , $I_{\mathrm{motor}}$

36:  **if** $\left(I_{\mathrm{motor}}\;\geq \;I_{\mathrm{motor}}^{TH}\right)$ **then**
37:   Decrease $P_{\mathrm{motor}}$ by δI

38:  **else**
39:   Store $P_{\mathrm{motor}}$ as the updated value
40:  **end if**
41:  **if** user interruption occurs **then**
42:   Break
43:  **end if**
44: **end while**

### 3.3 Detection algorithm

This section presents a slip detection algorithm that controls the grasping of each finger using tri-axial tactile feedback. The algorithm has been implemented and validated on a real-world robot setup, as shown in Figure 1. This method involves monitoring the force in the z-direction to ensure that the object remains engaged. Changes in resultant force within the xy-plane between consecutive readings serve as indicators of potential slip. When a slip is detected, commands are sent to the corresponding motor to adjust the gripping force accordingly. Moreover, a circuit protection mechanism is integrated to prevent electrical current overload in the motor. This integrated approach allows for calibration-free, real-time slip detection and correction across the fingers without prior knowledge of the properties of the object.

The pseudocode is shown in Algorithm 1, and was applied to each finger independently. The force readings from the tactile sensors were stored in the matrix $F^{\mathrm{raw}}$ with three column vectors $f_{x}^{\mathrm{raw}}$ , $f_{y}^{\mathrm{raw}}$ and $f_{z}^{\mathrm{raw}}$ , as shown in Equation 1, which at any iteration i

$$F^{\mathrm{raw}}\left[i\right]={\left[\begin{array}{ccc} f_{x}^{\text{raw}}\left[i\right] & f_{y}^{\mathrm{raw}}\left[i\right] & f_{i}^{\mathrm{raw}}\left[i\right] \end{array}\right]}^{⊤}$$ (1)

#### 3.3.1 Baseline forces acquisition phase

Before the program started, the first 20 sensor readings were taken and averaged to create the static baseline force vector $F_{0}$ , as shown in Equation 2

$$F_{0}=\frac{1}{N}\overset{19}{\underset{j=0}{\sum }}F^{\mathrm{raw}}\left[j\right]={\left[\begin{array}{ccc} f_{x,0} & f_{y,0} & f_{z,0} \end{array}\right]}^{⊤}$$ (2)

Also, the baseline xy-planar resultant force $f_{xy,0}$ was calculated by Equation 3

$$f_{xy,0}={\left\|t_{0}\right\|}_{2}=\sqrt{{\left(f_{x,0}\right)}^{2}+{\left(f_{y,0}\right)}^{2}},\;t_{0}={\left[\begin{array}{cc} f_{x,0} & f_{y,0} \end{array}\right]}^{⊤}$$ (3)

#### 3.3.2 Object engagement phase

Following the establishment of baseline force values, the object engagement phase was initiated. During this phase, contact detection was performed using closed-loop control, which involved continuous monitoring of $f_{z}^{\mathrm{control}}$ . At iteration k , the value was calculated by Equation 4

$$f_{z}^{\mathrm{control}}\left[k\right]=\left|f_{z}^{\mathrm{raw}}\left[k\right]-f_{z,0}\right|$$ (4)

Additionally, the motor position $P_{\mathrm{motor}}$ and the motor electrical current reading $I_{\mathrm{motor}}$ were also recorded. The finger was brought towards the object as long as $f_{z}^{\mathrm{control}}$ remained below the predefined detection threshold $f_{z}^{TH}$ , and $P_{\mathrm{motor}}$ was updated according to Equation 5

$$P_{motor}\left[k+1\right]=\left\{\begin{array}{cc} P_{\mathrm{motor}}\left[k\right]+\delta f_{z},\; & \text{if }f_{z}^{\mathrm{control}}\left[k\right]<f_{z}^{TH}\text{ and }P_{\mathrm{motor}}\left[k\right]<P_{\mathrm{motor}}^{TH}\text{ and }I_{\mathrm{motor}}\left[k\right]<I_{\mathrm{motor}}^{TH}\\ P_{\mathrm{motor}}\left[k\right],\; & \begin{array}{cc} & \;\text{if }f_{z}^{\mathrm{control}}\left[k\right]\geq f_{z}^{TH}\text{ and }P_{\mathrm{motor}}\left[k\right]<P_{\mathrm{motor}}^{TH}\text{ and }I_{\mathrm{motor}}\left[k\right]<I_{\mathrm{motor}}^{TH},\\ \; & \text{Object engagement phase completed.} \end{array} \end{array}\right.$$ (5)

where $\delta f_{z}$ was the motor increment by monitoring $f_{z}^{\mathrm{control}}$ , $P_{\mathrm{motor}}^{TH}$ was the motor position limit, and $I_{\mathrm{motor}}^{TH}$ was the limiting motor current threshold.

Furthermore, a termination condition was established, according to Equation 6, when

$$\text{Program terminated if}\left\{\begin{array}{c} P_{\mathrm{motor}}\left[k\right]\geq P_{\mathrm{motor}}^{TH},\text{ or}\;\\ I_{\mathrm{motor}}\left[k\right]\geq I_{\mathrm{motor}}^{TH}\; \end{array}\right.$$ (6)

Such a condition occurred when the sensor was misaligned with the target object. In such cases, adjustment of the gripping posture was necessary to ensure proper object handling.

#### 3.3.3 Slip detection phase

In the slip detection phase, the grasp was continuously stabilized while maintaining contact and ensuring the safety of the actuators within a closed-loop system. A three-iteration process was carried out within each loop. The first iteration focused on ensuring object engagement, during which Equation 5 was used to update $P_{\mathrm{motor}}$ .

The second iteration involved slippage detection, which was accomplished through continuous monitoring of the xy-planer resultant force $f_{xy}$ . From Equation 7, at iteration m

$$\begin{array}{ll} f_{xy}\left[m\right] & =\|t\left[m\right]{\|}_{2}=\sqrt{{\left(f_{x}^{\mathrm{raw}}\left[m\right]-f_{x,0}\right)}^{2}+{\left(f_{y}^{\mathrm{raw}}\left[m\right]-f_{y,0}\right)}^{2}},\\ & \;\;t\left[m\right]=\left[\begin{array}{c} f_{x}^{\mathrm{raw}}\left[m\right]-f_{x,0}\\ f_{y}^{\mathrm{raw}}\left[m\right]-f_{y,0} \end{array}\right] \end{array}$$ (7)

Also, ${\Delta f}_{xy}$ , which was the difference in $f_{xy}$ between consecutive readings, was calculated by Equation 8 as

$$\Delta f_{xy}\left[m\right]=\left\{\begin{array}{cc} \left|f_{xy}\left[m\right]-f_{xy,0}\right|,\; & m=0\\ \left|f_{xy}\left[m\right]-f_{xy}\left[m-1\right]\right|,\; & m>0. \end{array}\right.$$ (8)

In this work, $\Delta f_{xy}$ is used as a conservative “loss-of-stability” signal. Large, abrupt changes in tangential force may correspond to incipient slip, micro-slip, or sudden tangential disturbances. The controller is designed to respond to these events in order to detect slippage and minimize the risk of dropping the object. Because corrective actions are localized to the offending finger and bounded by motor-position and current limits, occasional conservative triggers do not result in uncontrolled increases in grasp force.

If ${\Delta f}_{xy}$ exceeded a predetermined threshold $\Delta f_{xy}^{TH}$ , a potential slipping event was identified. Therefore, $P_{\mathrm{motor}}$ was updated according to Equation 9

$$P_{\mathrm{motor}}\left[m+1\right]=\left\{\begin{array}{cc} P_{\mathrm{motor}}\left[m\right]+\delta f_{xy},\; & \text{if }\Delta f_{xy}\left[m\right]>\Delta f_{xy}^{TH}\text{ and }P_{\mathrm{motor}}\left[m\right]<P_{\mathrm{motor}}^{TH}\\ P_{\mathrm{motor}}\left[m\right],\; & \text{if }\Delta f_{xy}\left[m\right]\leq \Delta f_{xy}^{TH}\text{ and }P_{\mathrm{motor}}\left[m\right]<P_{\mathrm{motor}}^{TH} \end{array}\right.$$ (9)

where $\delta f_{xy}$ was the motor increment by monitoring ${\Delta f}_{xy}$ . Therefore, the motor was rapidly adjusted to increase finger bending, intended to boost gripping force and improve frictional engagement, thereby helping prevent slipping. Also, ${\Delta f}_{xy}$ is computed from differences between consecutive resultant tangential readings referenced to a runtime no-slip baseline. It does not require friction-parameter identification, or object- or pose-specific calibration procedures. In this study, no additional calibration performed by the user was necessary beyond the provided force output from the sensor.

The final iteration was the circuit protection, in which the motor position was adjust by monitoring $I_{\mathrm{motor}}$ . According to Equation 10, at iteration n

$$P_{\mathrm{motor}}\left[n+1\right]=\left\{\begin{array}{cc} P_{\mathrm{motor}}\left[n\right]-\delta I,\; & \text{if }I_{\mathrm{motor}}\left[n\right]\geq I_{\mathrm{motor}}^{TH},\\ P_{\mathrm{motor}}\left[n\right],\; & \text{otherwise} \end{array}\right.$$ (10)

where δI was the motor decrement by monitoring $I_{\mathrm{motor}}$ . Therefore, a secure grasp was maintained while the mechanism remained protected from damage caused by overcurrent.

### 3.4 Object engagement detection

Three objects with different rigidity, weight, and surface textures were selected for this study, as shown in Table 1. As mentioned in Section 3.1, the orientations of the sensors and the object during the grasping process were not guaranteed to be parallel. To mitigate this issue and ensure a consistent initial contact, a fixed value for $f_{z}^{TH}$ was empirically determined for all fingers before all experiments. Here, a monotonic closing motion was commanded to all fingers, in which all $P_{\mathrm{motor}}$ were increased from zero to the maximum motor, while $f_{z}^{\mathrm{control}}$ was logged. The minimum value at which contact was reliably detected across trials was then determined by visual inspection using a trial-and-error method, starting from a value of 50 mN with an increment of 50 mN per trial. The results were illustrated in Table 2, which found that the same $f_{z}^{TH}$ value was sufficient for all fingers to touch these objects successfully. This threshold was selected once for the platform and then kept unchanged for all fingers and objects in this study.

**TABLE 1:** Objects used in experiments.

Object	**Aluminium rod**	**Sponge**	**Water bottle**
Object image	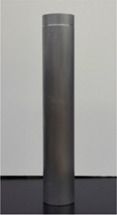	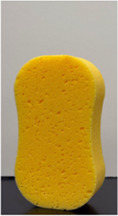	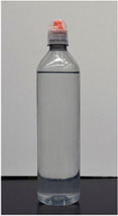
Size (mm)	Ø 48×275	55×120× 220	Ø 65×270
Weight (g)	380	41	745

**TABLE 2:** Parameter settings used in experiments.

Parameter	$f_{z}^{TH}$	$\Delta f_{xy}^{TH}$	$I_{\mathrm{motor}}^{TH}$	$\delta f_{z}$	$\delta f_{xy}$	δI	$P_{\mathrm{motor}}^{TH}$
Value	100	10	600	5	50	20	4,095

**FIGURE 3:**
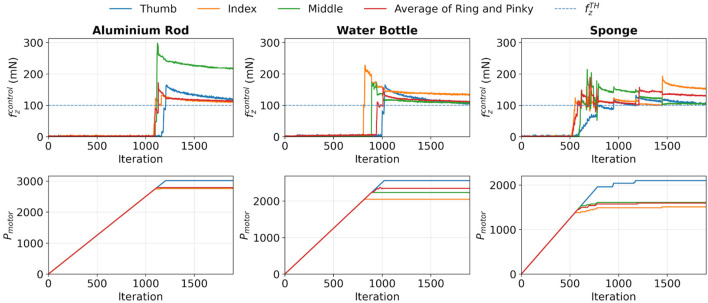
Evaluation of the applicability of $F_{z}^{TH}$ , which is illustrated as blue dashed lines in the figure, used in the algorithm for the object engagement test.

After determining $f_{z}^{TH}$ , this value was plugged into the algorithm, and the object engagement test was carried out to test its validity. The results are shown in Figure 3. The same $f_{z}^{TH}$ value was used in all subsequent experiments. Also, the maximum and final forces after stabilizing for each finger were listed in Table 3. From these results, a few observations were obtained. First, $f_{z}^{\mathrm{control}}$ slightly exceeded $f_{z}^{TH}$ after touching. The difference was caused by the fixed step increment in the motor control, which resulted in stepwise motion, introducing a small overshoot in $f_{z}^{\mathrm{control}}$ . Also, it was observed that the sensor values dropped after reaching their maximum values. It was mainly caused by the restoration of the shape of the elastomeric layer in tactile sensors. Moreover, if the object was soft, such as a sponge, the fluctuations were found to be even more rigorous than those of rigid objects, which was mainly due to the shape restoration of the soft object.

The results demonstrated that all motors could be effectively controlled using feedback from corresponding sensor readings, and their positions were maintained once $f_{z}^{TH}$ was reached. This showcased good conformability to different objects using the proposed method.

**TABLE 3:** Variation in z-directional forces of each finger when engaging with different objects.

Object	Aluminium rod				Water bottle				Sponge			
Finger	Thumb	Index	Middle	Average of ring and pinky	Thumb	Index	Middle	Average of ring and pinky	Thumb	Index	Middle	Average of ring and pinky
Maximum $f_{z}$ reading (mN)	164	133	298	172	165	228	175	157	132	193	214	189
$f_{z}$ reading after stabilizing (mN)	119	111	215	112	102	133	106	112	104	151	104	129

### 3.5 Slip detection on different objects

**FIGURE 4:**
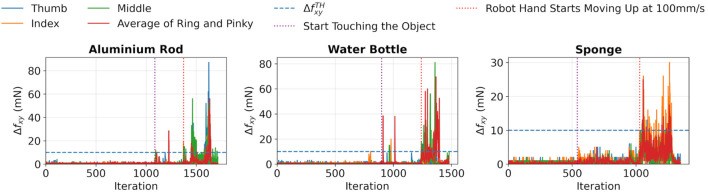
Results of the slipping test to estimate $\Delta F_{xy}^{Th}$ of different objects, in which the value is illustrated as blue dashed lines in the figure.

After finding $f_{z}^{TH}$ , the slip-detection algorithm was implemented to evaluate its responsiveness and robustness. Two parameters were required to be determined for the deployment of this algorithm. The first was $\Delta f_{xy}^{TH}$ , which was used to determine whether a slip event occurred or not. Here, a slipping test was implemented to evaluate the value. After gently touching the object, the hand was moved upward at a speed of 100 mm/s to evaluate $\Delta f_{xy}$ of the tested objects under slipping conditions. The results are shown in Figure 4, which clearly demonstrates that there was a rigorous fluctuation in $\Delta f_{xy}$ when slipping occurred. This finding provided strong evidence in support of the proposed slip detection method. This parameter required a balance on sensitivity. If $\Delta f_{xy}^{TH}$ was set too high, slip events went undetected, leading to false-negative outcomes. Conversely, if $\Delta f_{xy}^{TH}$ was set too low, the detector was vulnerable to mechanical vibrations and sensor noise, resulting in false positive results. From the results, it was shown that a single value for $\Delta f_{xy}^{TH}$ was efficient for all tested objects, as depicted as blue dashed lines in Figure 4. The second was $P_{\mathrm{motor}}^{TH}$ . As the grasping force increased with finger bending, this value directly influenced the maximum gripping force exerted on the object. For rigid or semi-rigid objects, a larger threshold was deemed acceptable since these objects could sustain a relatively greater gripping force without damage. Conversely, for soft objects, such as the sponge considered in this study, the threshold was restricted to prevent excessive deformation or damage of the object while ensuring grasp stability. The values for $\Delta f_{xy}^{TH}$ , $P_{\mathrm{motor}}^{TH}$ , and other parameters required for the algorithm are listed in Table 2. Unless mentioned otherwise, the values in the table were used throughout the study.

**FIGURE 5:**
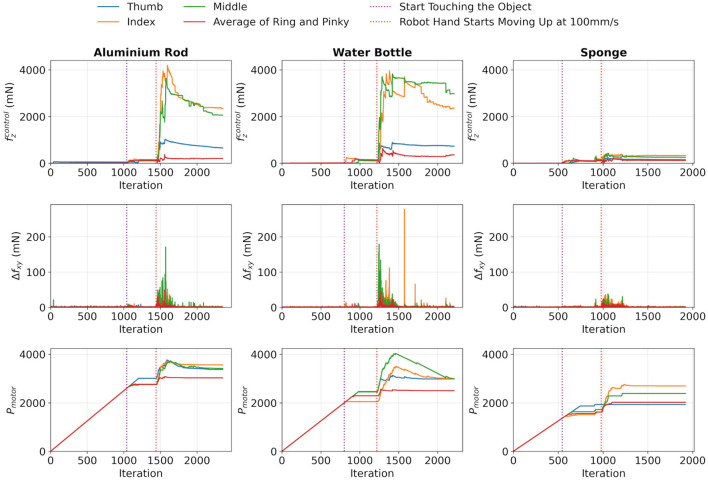
Results of the slip detection test of the three objects.

Then the slip detection test was carried out, and the results are shown in Figure 5. Also, the force variations and response time were shown in Table 4. From the results, it was shown that slippage could be successfully detected through monitoring $\Delta f_{xy}$ . When there was an abrupt change in $\Delta f_{xy}$ , the motor responded quickly by increasing $P_{\mathrm{motor}}$ to increase the gripping force, which was evidenced by the sudden increase in $f_{z}^{\mathrm{control}}$ and the elimination of $\Delta f_{xy}$ . As a result, the gripping force was increased significantly to stop the slippage from continuing. Additionally, it was observed that all motors were halted promptly once the slippage was eliminated. On the other hand, there were occasional decreases in $P_{\mathrm{motor}}$ , which resulted from the circuit protection mechanisms designed to prevent overcurrent and protect the circuit. Furthermore, the motor response time shows that the proposed algorithm responds quickly once a slip event is identified.

**TABLE 4:** Variation in forces and the response time of each finger with different objects during slip detection control.

Object	Aluminium rod				Water bottle				Sponge			
Finger	Thumb	Index	Middle	Average of ring and pinky	Thumb	Index	Middle	Average of ring and pinky	Thumb	Index	Middle	Average of ring and pinky
Maximum $f_{z}$ (mN)	1,028	4,195	3,640	372	891	3,956	3,829	630	226	424	426	226
Time to reach maximum $f_{z}$ (s)	2.26	2.88	2.50	2.11	3.81	3.06	3.82	1.31	2.81	2.77	3.09	4.22
$f_{z}$ reading after stabilizing (mN)	655	2,348	2060	203	732	2,365	2,984	361	150	332	257	124
Maximum variation in $\Delta f_{xy}$ (s)	42	61	171	50	32	278	179	45	12	36	38	24
$\Delta f_{xy}$ reading after stabilizing (mN)	0	0	1	1	1	1	0	1	1	1	1	0
Response time for motor once slip occur (s)	0.11	0.13	0.05	0.05	0.06	0.18	0.03	0.30	0.02	0.06	0.18	0.14

Therefore, slippage was successfully stopped on all tested objects by controlling $P_{\mathrm{motor}}$ based on sensor readings. This quick response ensured that no excessive force was applied to the objects, thereby minimizing the risk of damage and highlighting the effectiveness of the detection method used.

### 3.6 Slip detection at different lifting speed

**FIGURE 6:**
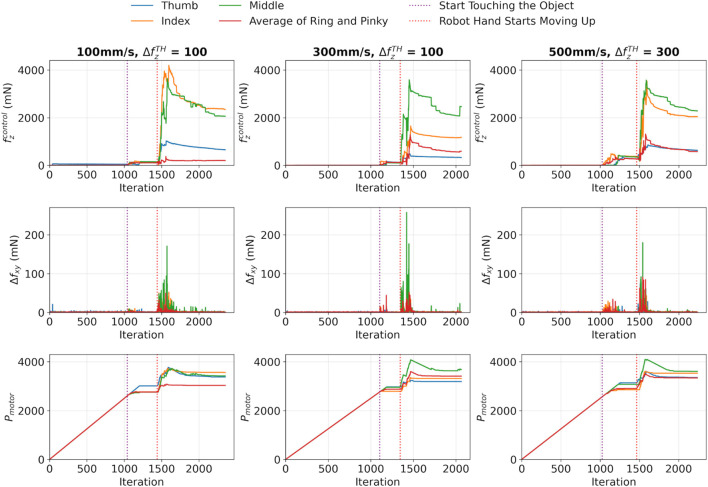
Slip detection on an aluminium rod at varying lifting speeds.

The detection algorithm was also evaluated against different lifting speeds. In this study, an aluminium rod was lifted at three vertical speeds: 100 mm/s, 300 mm/s, and 500 mm/s. The results are illustrated in Figure 6. Also, the force variations and response time were shown in Table 5. It was found that for vertical speeds of 100 mm/s and 300 mm/s, the $f_{z}^{TH}$ defined in Section 3.4 was sufficient to detect slippage. However, when the vertical speed increased to 500 mm/s, $f_{z}^{TH}$ needed to be increased slightly to 300 to stop slipping successfully. It was found that as the vertical speed increased, a lower $f_{z}^{TH}$ could still allow for slip detection, but the response time was not fast enough for the hand to make the necessary corrections. This delay was attributed to the sampling speed of the sensors and the response of the motors during each iteration of the slip-detection phase. Therefore, it was necessary to increase $f_{z}^{TH}$ to compensate for this issue. This change reflects a control setpoint adjustment for higher speed operation, and it does not involve any additional sensor calibration.

**TABLE 5:** Variation in forces and the response time of each finger with aluminium rod at different lifting speeds during slip detection control.

Lifting velocity	100 mm/s				300 mm/s				500 mm/s			
Finger	Thumb	Index	Middle	Average of ring and pinky	Thumb	Index	Middle	Average of ring and pinky	Thumb	Index	Middle	Average of ring and pinky
Maximum $f_{z}$ (mN)	1,028	4,195	3,640	372	485	1,651	3,589	1,281	858	3,531	3,577	1,313
Time to reach maximum $f_{z}$ (s)	2.26	2.88	2.50	2.11	2.06	2.30	1.93	2.18	2.27	1.99	1.80	1.64
$f_{z}$ reading after stabilizing (mN)	655	2,348	2060	203	332	1,178	2,462	593	628	2042	2,283	579
Maximum variation in $\Delta f_{xy}$ (s)	42	61	171	50	57	60	258	52	108	110	180	91
$\Delta f_{xy}$ reading after stabilizing (mN)	0	0	1	1	1	1	1	1	0	1	0	1
Response time for motor once slip occur (s)	0.11	0.13	0.05	0.05	0.19	0.14	0.02	0.14	0.05	0.15	0.02	0.03

The results showed that, despite varying lifting speeds, $P_{\mathrm{motor}}$ remained consistent. The robot hand applied a similar gripping force on the aluminium rod in each case. This confirms the effectiveness of closed-loop force control for slip detection, which proved reliable regardless of the lifting speed.

### 3.7 Sudden external disturbance detection

**FIGURE 7:**
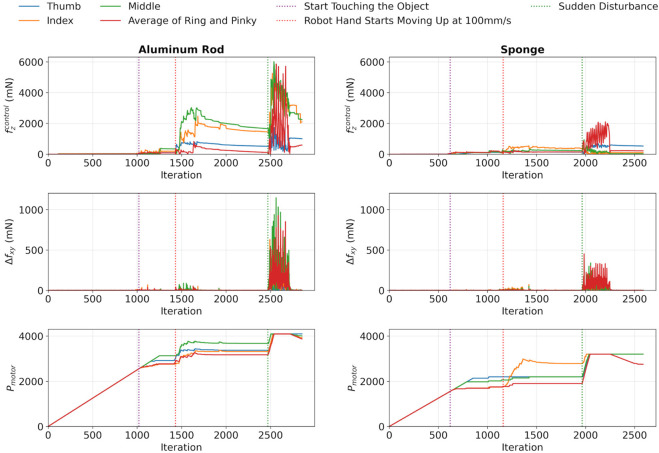
Test for sudden external disturbance to aluminium rod and sponge.

The robustness of the algorithm to sudden external disturbances was also evaluated in this study. After completing the slip-detection and establishing a stable grasp, the object was held in a stationary position. A manual pull was then applied vertically to simulate a sudden external disturbance. The results, shown in Figure 7, illustrated the response following the vertical lifting.

It was observed that this sudden pull induced a rapid fluctuation in $\Delta f_{xy}$ . In response to these abrupt changes, $P_{\mathrm{motor}}$ of all fingers were quickly adjusted to accommodate them. This adaptation resulted in an immediate increase in gripping force, which was indicated by the sudden increase in $f_{z}^{\mathrm{control}}$ . Furthermore, after the external disturbance was removed, some motors showed a decrease in $P_{\mathrm{motor}}$ as a result of the circuit protection implemented within the algorithm while maintaining the gripping force.

Overall, the test results verified the robustness of the proposed algorithm against sudden external disturbances, as well as its ability to maintain a safe and stable grasp after the disturbance was removed.

### 3.8 Discussion

#### 3.8.1 Significance of the proposed control method

A comparison of existing slip-detection approaches with our proposed controller is presented in Table 6. The comparison, along with the experimental results, demonstrates that our control strategy, using simple tri-axial piezoresistive sensors, achieves simple, robust, and responsive slip detection in an anthropomorphic hand. By relying on $f_{z}$ for object engagement and $\Delta f_{xy}$ for slip detection, the proposed method avoids the need for object- or pose-dependent calibration beyond the sensor provided force output and the runtime baseline acquisition, explicit friction modelling, or data-intensive learning pipelines, while still operating effectively across objects with distinct rigidity, weight, and surface textures.

**TABLE 6:** Comparison of piezoresistive slip-detection methods.

Reference	Sensor arrangement	Availability	Slip detection theory	Strategy	Calibration^a^
Zhao et al. (2025)	Array	Commercial	CNN slip classifier	Hybrid	No
Chen et al. (2025)	Single	Commercial	CNN on tactile signals	Hybrid	No
Chen et al. (2024)	Single	Lab-made	Shear/vibration sliding cues	Control	No
Liu and Howe. (2023)	Single	Commercial	Probabilistic friction/slip	Learning	Yes
Kim et al. (2022)	Single	Lab-made	Normal vs. shear thresholds	Control	Yes
Mo et al. (2022)	Array	Lab-made	Piezoresistive and inductive slip cues	Control	Yes
Pei et al. (2021)	Array	Lab-made	Spatiotemporal force pattern	Control	No
Wang et al. (2019)	Array	Lab-made	DWT on 3-axis forces	Control	Yes
Van Wyk and Falco. (2018)	Single	Commercial	LSTM on tactile time series	Hybrid	Yes
Deng et al. (2017)	Single	Commercial	Torque/stiffness reflex	Control	No
Stachowsky et al. (2016)	Single	Lab-made	Force/vibration slip signal	Control	Yes
Zhang et al. (2015)	Array	Lab-made	Friction-cone grasp stability	Control	Yes
Our approach	Single	Commercial	Planar tangential force variation	Control	No

^a^ Calibration denotes an explicit procedure performed before or during deployment to obtain sensor force mapping coefficients or to identify physics model parameters such as friction, beyond manufacturer-provided sensor outputs and the runtime baseline acquisition.

In the proposed controller, calibration-free means that no additional user-performed per-sensor or per-object calibration was conducted, and the slip decision relies on the runtime baseline and consecutive changes rather than fitted friction parameters. A key advantage of the proposed algorithm is that corrective actions are localized, and commands are sent only to the motor whose corresponding finger detects a slip, thereby increasing gripping force. This approach reduces unnecessary force exerted on fingers in no-slipping conditions, improving grasp efficiency and potentially reducing wear on both sensors. Additionally, the experimental results indicate that a compact set of threshold parameters $f_{z}^{\text{TH}}$ and $\Delta f_{xy}^{\text{TH}}$ for all fingers is adequate to generalize across a variety of everyday items. Moreover, the experimental results under varying lifting speeds and sudden external disturbances further indicate that the closed-loop scheme can stabilize grasps in dynamic conditions without requiring extensive tuning.

#### 3.8.2 Limitations and future research

Despite the advantages of the current method, several limitations remain. In this article, calibration-free indicates that the controller does not need object or pose-specific tuning or friction parameter identification. It relies on a runtime baseline and changes in force rather than fitted models. First, the method assumed a fixed hand posture during grasping because the wrist DoFs were fixed. The test objects were positioned to ensure successful grasps, thereby avoiding misalignment issues. Second, the thresholds $f_{z}^{\text{TH}}$ and $\Delta f_{xy}^{\text{TH}}$ were selected offline using iterative testing. This approach may limit adaptability across a broader range of objects and tasks. These threshold values were kept fixed throughout the experiments, except for the highest lifting speed condition discussed in Section 3.6, where $f_{z}^{\text{TH}}$ was increased due to sampling and motor response limits. Third, the current implementation was constrained by the sampling rate of the tactile sensors and the response time of the actuators, which became critical at higher lifting speeds, where higher thresholds were deemed necessary to compensate for the limited temporal resolution. This increase should be interpreted as a control setpoint adjustment for faster dynamics rather than an additional sensor calibration step. Finally, the experimental validation focused only on vertical lifting and disturbances using three representative objects. More complex in-hand manipulation, lateral perturbations, and cluttered environments were not considered in this study.

These limitations motivate several potential directions for future research. One approach is to develop an adaptive thresholding scheme that adjusts $f_{z}^{\text{TH}}$ and $\Delta f_{xy}^{\text{TH}}$ in real time based on observed force and slip history or estimated object properties. This adjustment would improve robustness across different tasks and hardware variations. Another direction is to integrate a camera into the system to incorporate vision in conjunction with the tactile-based controller. This integration can help actively refine grasp posture and contact placement, reducing the likelihood of misalignment and improving coverage of complex object geometries. Additionally, learning-based components, such as reinforcement learning, could be incorporated into the existing rule-based framework to modulate thresholds while preserving real-time operation without object- or pose-specific calibration or friction parameter identification.

## 4 Conclusion

This article introduces a calibration-free, per-finger force-feedback slip controller for an anthropomorphic hand that employs tri-axial tactile sensing and simple thresholding for real-time slip detection and correction on unknown objects. The design allows for localized adjustments for each finger, enhancing grasp efficiency and reducing unnecessary force on stable contacts. Experiments demonstrate rapid responses and stable grasps with controlled grip force across various object types and conditions, demonstrating the effectiveness of tri-axial sensing for slip control without calibration. However, limitations include a fixed hand posture due to reduced degrees of freedom, reliance on offline-tuned thresholds, and validation on a limited range of tasks. Future work will explore adaptive thresholding, vision-based grasping enhancements, and learning methods to improve performance on unseen objects and tasks while maintaining the framework’s simplicity and real-time functionality.

## Data Availability

The original contributions presented in the study are included in the article/Supplementary Material, further inquiries can be directed to the corresponding author.
